# Research Note: Effect of corn silage and alfalfa meal as alternative induced molt methods to improving *Salmonella Enteritidis* resistance in laying hens

**DOI:** 10.1016/j.psj.2022.101984

**Published:** 2022-06-02

**Authors:** Hossein Ayasi, Behrouz Dastar, Taghi Ghoorchi, Seyed Reza Hashemi, Alijan Tabaraei, Morteza Alemi

**Affiliations:** ⁎ Department of Animal and Poultry Nutrition, Faculty of Animal Science, Gorgan University of Agricultural Sciences and Natural Resources, Gorgan, Iran; † Department of Animal and Poultry Physiology, Faculty of Animal Science, Gorgan University of Agricultural Sciences and Natural Resources, Gorgan, Iran; ‡ Department of Microbiology and Immunology, Golestan University of Medical Sciences, Gorgan, Iran

**Keywords:** corn silage, laying hen, molting, *Salmonella Enteritidis* resistance

## Abstract

This experiment was conducted to evaluate diets containing a high level of corn silage and alfalfa meal in inducing molt and reducing susceptibility to *Salmonella Enteritidis* (**SE**) colonization in laying hens. Thirty-two healthy hens were examined by cloacal swab samples to be free of *Salmonella.* Then they were weighed individually and distributed to 4 experimental groups containing 8 hens each, including Full-fed (control, **FF**); total feed withdrawal (positive control for molt induction, **FW**); 80% corn silage (**CS**) + 20% layer diet (CS80), and 80% alfalfa meal (AM) + 20% layer diet (AM80). The molting program was initiated at 71 wk of age. On d 4 of the experiment, all hens were inoculated with SE by oral gavage. All hens were first weighed at the ending molting period on d 10 and then euthanized by CO2 gas. The internal organs including the ovary, oviduct, liver, and spleen, were excised aseptically and weighed. Cloacal swab and feed samples at the beginning and organ samples (liver, ovary, spleen, and cecum) were collected from each hen at the end of the experiment and examined for SE colonies. Molted birds lost roughly 14 to 27%t of their body weight and had significantly lower organ weight and egg production compared to FF group (*P* < 0.05). No significant difference was observed in the number of days to zero egg production between molted treatments. The SE positive organs did not significantly differ between CS80 and AM80 with FF treatment. Treatment CS80 had the lowest crop pH and differed substantially from treatment FW. In conclusion, results indicate that using corn silage and alfalfa meal, can improve resistance to salmonella Enteritidis during molt inducing compared to traditional feed withdrawal.

## INTRODUCTION

At the end of the laying cycle, egg production, and quality decline significantly, leading producers to induce molt for improving performance. Molt induction is an effective tool for the economic management of laying flocks to prolong the reproductive period and improve the egg quality of aged hens. Typical molting programs involve reducing light hours and removing feed until body weight losses roughly more than 25% and egg production cease. However, feed withdrawal as a method for inducing molt has drawn criticism due to animal welfare and food safety concerns. It has been reported that feed withdrawal is a stressful conventional method that can impair laying hens' immunological function and alter the gut microbiota population. These circumstances increase the susceptibility of layers to Salmonella infection and, therefore, more readily transmitte the organism to other uninfected hens in the flock (Ricke., 2003). The high incidence of salmonellosis infections in molted hens is still an important health challenge because it negatively affects the flock's health and is a significant source of foodborne gastroenteritis in humans.

*Salmonella Enteritidis* infection is usually marked by increased intestinal shedding and colonization in internal organs such as the liver, spleen, and ovaries. The ceca and the crop are the main sites of Salmonella colonization in laying hens. These parts of gastrointestinal tract (**GIT**) contain high short-chain fatty acids (**SCFAs**; lactate, acetate, propionate, and butyrate) produced by the indigenous fermentative microflora. However, it was shown that conditions that reduce fermentation activity and finally decrease microflora in the GIT could facilitate *Salmonella Enteritidis* colonization (Ricke, 2003). The SCFAs have been extensively examined as potential modulators for Salmonella virulence after they were shown to change GIT environment acid to unsuitable for salmonella species. Over the last few years, the ad libitum feeding of alfalfa meal (Donalson et al., 2005) and corn silage (Ayasi et al., 2016) were identified as effective methods for molt induction.

Corn silage (Zea mays) is a fermented feed since it has an optimum level of SCFA. Moreover, corn silage is rich in indigestible plant fibers feedstuff for poultry. Accordingly, the objective of the current study was to explore the efficacy of diets containing a high level of corn silage in inducing molt and reducing susceptibility to *Salmonella Enteritidis* colonization compared to other conventional methods in laying hens.

## MATERIALS AND METHODS

### Experimental Design and Molting Procedure

The Animal Ethics Committee approved the experimental protocols of Gorgan University of Agricultural Sciences and Natural Resources, Gorgan, Iran. Commercial Hy-Line White Leghorn hens 70 wk old were obtained from a local commercial layer farm. The hens were placed in the wire cage system under controlled climate conditions in the poultry station of Gorgan University of Agricultural Sciences and Natural Resources. Hens were allowed a week for acclimatization on a 16L: 8D lighting program before the molting period. During this time, they were fed a commercial layer diet and allowed full access to feed and water. Thirty-two healthy hens which were negative for salmonella after examining by cloacal swab samples were weighed individually and distributed to 4 experimental groups containing 8 hens each. The hens of the first group were full-fed (**FF**) with the commercial layer diet throughout the molting period, while the second group feed withdrawal (**FW**). The third and fourth groups full-fed with a feed mixture of 80% corn silage and 20% the commercial layer diet (CS80) or 80% alfalfa meal and 20% the commercial layer diet (AM80), respectively. The commercial layer diet consisted of 32.4% corn, 18.7% soybean meal, 28% wheat, 4.52% meat meal, 5% commercial concentrate, 3.3% oyster shell, 6.1% calcium carbonate, 0.18% salt, 1.8% alfalfa meal, and contained 2,750 kcal/kg metabolizable energy,15.5% crude protein, 4.31% calcium, and 0.42% available phosphorous.

To calculate body weight change and measure feed intake, hens were weighed at the starting and ending molting period at 10 d. During molting period, all hens had free access to water, and the light program was changed to an 8L: 16D photoperiod. Before preparing, the feeds were examined for salmonella, and salmonella was not detected in the feed samples. Corn silage was chopped and mixed daily with the commercial diet. On d 4 of the molting, all hens were inoculated with *Salmonella Enteritidis* by oral gavage. On d 10 of the study, all hens were euthanized by CO_2_ gas, and the ovary, oviduct, liver, and spleen were excised aseptically and weighed.

### Salmonella Isolate and Sample Collection

The bacterial strain (*Salmonella enteritidis*), which was used in this experiment, was obtained from the Faculty of Veterinary Medicine, University of Tabriz (Tabriz, Iran), and then confirmed with a biochemical test. The challenge inoculum was obtained from an overnight culture that had been previously transferred 3 times in trypticase soy broth. The culture was serially diluted in sterile phosphate-buffered saline to a concentration of approximately 5.6 × 10^4^ CFU/mL.

Cloacal swab and feed samples at the beginning and organ samples (liver, ovary, spleen, and cecum) were collected from each hen at the end of the experiment incubated for 24 h at 37°C in tetrathionate broth. After incubation, the broth was streaked onto brilliant green agar plates, incubated for an additional 40 h at 37°C, and examined for the presence of SE colonies (Andreatti Filho et al., 2019). Suspect colonies were confirmed biochemically.

The crop pH was determined by inserting a sterile glass pH electrode through an incision in the crop wall, ensuring that the electrode remained in contact with the crop mucosal surface.

### Statistical Analyses

Data were subjected to one-way analysis of variance (**ANOVA**) using GLM procedure of SAS software (SAS Institute Inc., Cary, NC, 2005). When the influence of treatment was stated by ANOVA test, Tukey's test was used to estimate significant differences among treatment means. Treatment means differed significantly at *P* < 0.05. The total *Salmonella enteritidis*-positive numbers of organs from each treatment and all hens were compared by Pearson's χ2 test (SAS Institute Inc., Cary, NC, 2005). Fisher's exact test was used to determine differences between treatment groups.

## RESULTS AND DISCUSSION

### Body Weight and Feed Intake

The effect of treatments on body weight (**BW**) and feed intake is shown in Table 1. All hens in molting groups lost a part of their body weight. Meanwhile, BWL was lesser in AM80 (14.39%) and CS80 (17.74%) groups compared to FW group (26.87%) (*P* < 0.05). This event is natural because if we ignored alfalfa meal and corn silage nutrients, the hens in these 2 groups still received a 20% egg layer diet, which provided a part of their body requirements. The value of 11 to 25.1% BWL during molting period was reported (Donalson et al., 2005) due to the use of diet with different levels of alfalfa meal. Ayasi et al. (2016) also reported that feeding corn silage from 70 to 100% levels for 10 d caused hens to lose 29.31 to 34.17 % of their BW. Anyway, body weight loss during the molting period is one of the most important factors directly related to post-molt egg production and eggshell qualities. The first visible change during induce molting is feed intake reduction. Feed intake was significantly lower in hens who received CS80, or AM80 treatments than in the FF group (*P* < 0.05), and this decrease was more significant for AM80 than CS80. Hens intake lesser feed during molting period rather than normal rearing conditions because of decreasing feeding stimulation with reduced daylight hours. Use of appetite suppression agents and high fiber feedstuffs causes to slow passage rate in the guts and might have influenced feed intake, causing a feeling of satiety and thus refraining hens from eating. Corn silage and alfalfa meal contain high levels of fiber and are known as bulky feeds, which may reduce the hens feed intake by decreasing palatability (Ayasi et al., 2016; Chanaksorn et al., 2019).

**Table 1:** Body weight loss (BW), feed intake, relative organ weight, egg production, and days to zero egg production of laying hens molted by various methods.

Treatments^1^	% BW lose^2^	Feed intake (g/hen/d)	% Organ weight				% Egg production	Days to 0 eggs
			Oviduct	Ovary	Liver	Spleen		
FF	+5.06^c^	83.31^a^	3.40^a^	2.65^a^	3.23^a^	0.13	54.16^a^	-
FW	26.87^a^	-	1.43^b^	0.70^b^	2.38^b^	0.15	22.22^b^	3.75
CS80	17.74^b^	29.40^b^	1.15^b^	0.66^b^	2.11^b^	0.15	19.44^b^	3.25
AM80	14.39^b^	16.62^c^	1.12^b^	0.55^b^	2.19^b^	0.16	13.88^b^	4.75
SEM	2.01	3.80	0.18	0.18	0.13	0.018	4.86	0.47
*P*-value	0.0001	0.0001	0.0001	0.0001	0.0001	0.71	0.0003	0.13

1 FF, full fed; FW, feed withdrawal; CS80, 80% corn silage and 20% layer diet; AM 80,–80% alfalfa meal and 20% layer diet; SEM, standard errors of mean.

2 Inital BW were 1,288.75, 1,300.25, 1,304.25, and 12,77.63 g for full feed (FF), feed withdrawal (FW), 80% corn silage (CS), and 80% alfalfa meal (AM), respectively that not have significant difference.

abc Means within a column with no common superscripts are significantly different (*P* < 0.05).

### Organs Weight

There was no found difference between molted treatments with different methods. Hens in control FF treatment had significantly higher internal organ weights, including ovary, oviduct, and liver weight, except spleen, rather than all molting treatments (Table 1, *P* < 0.05). The liver is a vital organ involved in carbohydrate, lipid, and protein metabolism and many other metabolic activities. Fasting chicks for extended periods resulted in decreased levels of liver glycogen, lipids, and protein which this depletion subsequently leads to decreased liver weight. Dastar et al. (2016) discussed forced molting accompanied by fasting hens for several days. Hence, regression of the liver is expected because of losing liver glycogen and lipids as energy sources. The regression of the ovary is essential to obtaining egg production and eggshell quality during the second production cycle because the loss of reproductive weight is linked to the overall rejuvenation process. The molted treatments induce ovarian regression, resulting in reduced synthesis of the steroid hormones (Chanaksorn et al., 2019). The ovarian regression would negatively affect pituitary gonadotrope responsiveness, causing a reduction of the steroidogenic capacity of ovarian follicles (Chanaksorn et al., 2019). That can decrease estrogen-dependent egg component synthesis in the liver and as a result in liver weight loss.

### Egg Production

The effect of treatments on hen-day egg production and the number of days to zero egg production are shown in Table 1. The highest egg production value belonged to FF group (54.16%), which was significantly greater than molted groups (*P* < 0.05). Egg production ceased completely in all molting treatments. However, there was no insignificant difference between molting groups for a mean number of days to zero egg production (Table 1). Hens stopped egg laying several days after molting because of receiving an inadequate amount of nutrients as shown by some researchers (Ayasi et al., 2016).

### *Salmonella Enteritidis* Invasion

The number of organs with SE culture-positive was significantly increased in the molted hens with no difference between molting methods (Table 2). The incidence of SE was greater in FW treatments than FF control, CS80%, and AM80% treatments. The main differences between treatments were found when all organ samples were summed, and the incidence of *Salmonella Enteritidis* colonization was analyzed. The FF treatment had lower total *Salmonella Enteritidis* positive numbers in the 4 tested organs than all experimental treatments, and the difference was only significant with the FW treatment.

**Table 2:** Invasion of Salmonella Enteritidis into organs and crop pH of laying hens induced to molt by various methods.

Treatments^1^	Liver	Ovary	Spleen	Seca	All organs	Crop pH
FF	0/6^b^	1/6^ab^	0/5^b^	2/5^b^	3/22^b^	5.79^b^
FW	4/6^a^	3/6^a^	5/6^a^	5/5^a^	17/23^a^	7.2^a^
CS80	0/6^b^	1/6^ab^	0/6^b^	4/6^ab^	5/24^b^	5.49^b^
AM80	1/6^ab^	0/6^b^	1/5^b^	4/5^ab^	6/22^b^	6.62^ab^
SEM						0.373
*P*-value	0.012	0.18	0.003	0.19	0.0001	0.002

1 FF, commercial layer diet; FW, total feed withdrawal; CS80, 80% corn silage and 20% layer diet; AM 80–80% alfalfa meal and 20% layer diet.

ab Means within a column with no common superscripts are significantly different (*P* < 0.05).

Contamination of egg products has been the most common way to transmit human foodborne illnesses, especially salmonellosis. Egg contamination usually occurs, followed by the invasion of this pathogen into the reproductive organs after the initial infection. Reducing the incidence of salmonella in the internal organs of hens could minimize the possibility of contamination of eggs used for human consumption.

High fiber content feed-stuffs have been shown to increase the transit time in the GIT of hens and thereby increase fermentable product substrates such as SCFAs. The results of studies showed that short-chain and medium-chain fatty acids have antibacterial effects, especially on Salmonella species (Durant et al., 2000). The impact of organic acids on suppressed Salmonella invasion can be explained by changes in the virulence genes expression of salmonella. On the other hand, corn silage is a fermentation feedstuff that contains high levels of SCFAs. The concentration of different forms of organic acids in corn silage depends on many factors. Still, corn silage in normal fermentation usually contains 4 to 7% lactic acid, 1 to 3% acetic acid, and ˂0.1% propionic acid (Kung et al., 2015).

These results indicated that corn silage could be an alternative method for molting and reduced susceptibility to *Salmonella Enteritidis*. There were many studies conducted for estimated organic acids surface changes during molt induction and some of the studies use organic acids in water for compensation fermentation reduction during inducing molt with feed withdrawal (Dunkley et al., 2007). It appears that using a fermentation feedstuff for inducing molt can be a suitable way for solving the problem.

### Crop pH

The FW treatment had higher crop pH values than all other treatments that were significant with FF and CS80 treatments (*P* < 0.05; Table 2). There was no difference between AM80 treatment and all other treatments. Feed withdrawal has been reported to be the main factor causing decreases in crop Lactobacilli populations that produce primarily lactic acid, thereby allowing crop pH to rise in hens (Corrier et al., 1999). The results showed that using corn silage can decrease crop pH due to having high levels of organic acids, even lower than FF hens. The crop is the first environment encountered by Salmonella after infection in poultry (Fuller, 1973).

## DISCLOSURES

The authors have no conflicts of interest to declare.

## References

[bib0001] Andreatti Filho R.L., Milbradt E.L., Okamoto A.S., Silva T.M., Vellano I.H.B., Gross L.S., Oro C.S., Hataka A. (2019). *Salmonella Enteritidis* infection, corticosterone levels, performance and egg quality in laying hens submitted to different methods of molting. Poult. Sci..

[bib0002] Ayasi H., Dastar B., Ghoorchi T., Hashemi S.R., Tabaraei A. (2016). Effect of utilization of maize silage in molt inducing diets on performance, immune response and bone quality in laying hens. J. Anim. Feed Sci..

[bib0003] Chanaksorn M., Boonkaewwan C., Kayan A., Gongruttananun N. (2019). Evaluation of molt induction using cassava meal varying the length of feeding period in older (90 week) laying hens. Poult. Sci..

[bib0004] Corrier D.E., Byrd J.A., Hargis B.M., Hume M.E., Bailey R.H., Stanker L.H. (1999). Presence of Salmonella in the crop and ceca of broiler chickens before and after preslaughter feed withdrawal. Poult. Sci..

[bib0005] Dastar B., Khosravi A., Boldajie F., Ghoorchi T. (2016). Effect of calcium with and without probiotic, lactose, or both on organ and body weights, immune response and caecal microbiota in molted laying hens. J Anim Physiol Anim Nutr..

[bib0006] Donalson L.M., Kim W.K., Woodward C.L., Herrera P., Kubena L.F., Nisbet D.J., Ricke S.C. (2005). Utilizing different ratios of alfalfa and layer ration for molt induction and performance in commercial laying hens. Poult Sci..

[bib0007] Dunkley K.D., McReynolds J.L., Hume M.E., Dunkley C.S., Callaway T.R., Kubena L.F., Nisbet D.J., Ricke S.C. (2007). Molting in Salmonella Enteritidis challenged laying hens fed alfalfa crumbles I. Salmonella Enteritidis colonization and virulence gene hilA response. Poult Sci..

[bib0008] Durant J.A., Corrier D.E., Ricke S.C. (2000). Short-chain volatile fatty modulate the expression of the hilA and invF genes of *Salmonella Typhimurium*. J. Food Prot..

[bib0009] Fuller R. (1973). Ecological studies on the Lactobacillus flora associated with the crop epithelium of the fowl. J. Appl. Microbiol..

[bib0010] Kung L.J., Lim J.M., Hudson D.J., Smith J.M., Joerger R.D. (2015). Chemical composition and nutritive value of corn silage harvested in the northeastern United States after Tropical Storm Irene. J. Dairy Sci..

[bib0011] Ricke S.C. (2003). The gastrointestinal tract ecology of Salmonella enteritidis colonization in molting hens. Poult. Sci..

